# Long Life

**Published:** 1859-11

**Authors:** 


### LONG LIFE.

Many young people feel as if very old age is a misfortune which is to be avoided, yet few become so old but that they would like to live a little longer. As in one sense of the word it is not with us to live or die, but to live in sickness and misery or in health and happiness to extreme old age, it is the dictate of true wisdom to begin early to live in the manner best calculated to secure a healthy old age, and to know what that way is, we cannot perhaps do better than to use the experience of the old, the healthful, and the wise. Within a few days, the venerable and lively Grant Thorburn writes, in sight of ninety:

"I am often asked how I live. Hoping it may induce some dyspeptic mortals to consider their ways and be healed, I give you the prescription.

"I rise at seven, A. M., wash, dress, and smoke a Dutch pipe-full of Miller's & Mickle's best tobacco; at eight, I breakfast on half a pint of coffee, bread, dried meat or fish; I dine at twelve; I never eat enough; I close dinner with half a pint of coffee and a crust of bread, smoke my pipe, and if the weather is warm, I lie on the sofa one hour; drink two cups of tea at five P. M.

"I never eat butter on my bread; I never put milk in either tea or coffee; these privations are no restraint; my stomach says "no." I use sugar in both tea and coffee; I never eat between meals; I drink between meals at least one quart of cold coffee per day, without either sugar or milk. Some one said tkat drinking coffee and smoking tobacco is a slow poison; in my case it has been slow indeed, for I have drank three pints of coffee, and have smoked five pipes of tobacco every day during the last fifty years, except when sea-sick in crossing the Atlantic. In September, 1798, when the yellow fever swept the streets of New York, I noticed that a chill always preceded the fever. By way of prevention, I put on woolen flannel, which I wore next my skin, from my ankles to my neck; my wife and children put on the same. In the seventeen summers during which the fever prevailed, I never left the city, and every year, more or less, I nursed among the sick, yet neither myself, my wife, nor any of my seven children ever caught the fever.

"From that day, I have contrived to wear the same flannel garments, and during all that period I have been only six days confined to the house (not bed) by indisposition. I never feel a head, heart, or rheumatic pain. The savory food in which I delighted sixty years ago, tastes as sweet to my mouth now as it did then. I never was drunk in my life; I sleep without rocking, walk without a staff, and ate without brandy or bitters

"Every day except Sunday, I cut wood for the stove with a buck and saw. The nursing care of my dear wife is now the rod and staff of my life."

If any of our readers should plead the example of our correspondent as their excuse for the use of coffee and tobacco, they should remember that he has lived long in spite of them, and without them would most probably have lived longer than he will do. At all events, let them couple with it his long life of regular, plain living, and honest industry. If he never got drunk, so must not they; if he never eat enough, so must not they; if he never exceeded in frequency and quantity, so must not they; if he failed not to maintain "a conscience void of offence toward God and toward men," so must not they; if they take a part of his habits, they must take them all. We have certainly never known a drunkard who did not love strong coffee and use tobacco largely, and these are the first steps toward a drunken, vicious, and wasted life, and if in any instance a coffee drinker and a tobacco user fails to land in the gutter, it is more of the grace of God than of the gift of man. If any person will promise never to taste a drop of spirituous liquors, we will give him an assurance and a permission—the assurance that he will never die a sot, and the permission to drink half a pint of moderately strong coffee at each meal—but never more, never oftener, never stronger.

